# Influence of Post-Processing by Annealing on Additive Manufacturing Productivity and Mechanical Properties in Three-Point Bending of Additively Manufactured Specimens from PETG and Recycled PETG

**DOI:** 10.3390/polym18192348

**Published:** 2026-09-26

**Authors:** Dragos Gabriel Zisopol, Mihail Minescu, Dragos Valentin Iacob, Bogdan Vasile Nastase

**Affiliations:** 1 Mechanical Engineering Department, Petroleum-Gas University of Ploiesti, 100680 Ploiesti, Romania; zisopold@upg-ploiesti.ro (D.G.Z.);; 2 Department of Mechanical Engineering, Doctoral School, Petroleum-Gas University of Ploiesti, 100680 Ploiesti, Romania; bogdan.nastase@upg-ploiesti.ro

**Keywords:** additive manufacturing, postprocessing, manufacturing parameters, annealing, optimization

## Abstract

Additive manufacturing technologies using extrusion of plastic material (MEX) stand out due to the accessibility of equipment and raw materials (filaments, granular materials), the variety of equipment and materials, low operating costs and the potential for use in non-industrial environments. However, additive manufacturing using extrusion of plastic material (MEX) faces a number of factors that reduce the mechanical characteristics of parts and the productivity of the manufacturing process, preventing the adoption of this manufacturing technology on a large scale. Post-processing is a crucial step in additive manufacturing using plastic extrusion, which has a significant impact on the finished product. In this sense, in this paper, the influence of the annealing heat treatment (temperature, t = 75 °C and duration, d = 180 min) on the mechanical characteristics during three-point bending of the specimens manufactured additively by extrusion of filaments made of PETG and recycled PETG 100 (rPETG 100, where 100 represents the amount of recycled material) is studied using the layer height deposited in one pass, L_h_ = (0.10/0.15/0.20) mm, and the percentage fill density, I_d_ = (50/75/100)%. The annealing heat treatment generated a 14.24% increase in flexural strength of the additively manufactured PETG specimens and a 2.48% increase in the flexural strength of the additively manufactured rPETG specimens.

## 1. Introduction

Given the need to transition to a circular economy in the plastics sector, the use of recycled polymers has become a priority for both academia and industry. Polyethylene terephthalate glycol (PETG) offers optimal mechanical properties, superior chemical resistance, and ease of processing, making PETG a material used in additive technologies through extrusion of plastic materials [1,2,3,4,5,6,7,8]. Due to the thermal degradation of the polymer chains following PETG reprocessing, its reuse generates challenges in terms of maintaining mechanical characteristics and safety in operation [9,10,11,12,13,14,15]. To achieve the desired mechanical properties in parts fabricated via material extrusion additive manufacturing, it is necessary to optimize the printing parameters, which have a major influence on the mechanical characteristics, operational behavior and productivity [16,17,18,19,20,21,22,23]. Within the manufacturing processes, productivity is a crucial performance indicator, and unlike subtractive and formative manufacturing, additive manufacturing technologies have a low productivity [24,25,26,27,28]. In this context, in order to increase the productivity of additive manufacturing processes and improve the mechanical characteristics of parts, it is recommended to use post-processing by heat treatment (annealing), this type of heat treatment facilitating the relaxation of stresses during the manufacturing process and improving the degree of molecular cohesion [29,30,31,32,33,34]. In [35], a technical–economic study on the optimization of material extrusion (MEX) parameters is presented with scope of the manufacture of tensile and compression specimens from PETG and ASA. The conclusions show that for tensile specimens from PETG, the dominant parameter is the height of the layer deposited in one pass, and for compression specimens made from PETG, the dominant parameter is the filling percentage. In the case of tensile and compression specimens made from ASA, the dominant parameter is the filling percentage. The optimal manufacturing parameters identified are layer height 0.20 mm and filling percentage 100%. In [36], the influence of additive manufacturing via plastic extrusion parameters (layer height, filling density, number of perimeters) on the mechanical and qualitative performance of parts manufactured from PLA is investigated. The conclusions of the study show that the impact resistance is influenced by the considered parameters, and the surface roughness is influenced by the layer height deposited in one pass. The optimal parameters identified by the Grey Relational Analysis (GRA) method are layer height 0.30 mm, filling density 10% and 6 perimeters. Paper [37] presents the results of the research on the effect of parameters additive manufacturing by material extrusion (layer thickness, nozzle temperature, printing speed and deposition orientation) on the flexural strength, density and printing time of parts made of PLA reinforced with short carbon fibers (PLA-CF). The results of the study show that of the four parameters considered, orientation is the main parameter influencing the flexural strength, and the layer thickness is the parameter with secondary influence. The optimal parameters identified are represented by a layer thickness of 0.10 mm, nozzle temperature of 210 °C, printing speed of 30 mm/s and deposition orientation of 0°. The study presented in [38] investigates the optimization of MEX parameters (layer height, filling density, extrusion temperature, layer orientation) on the flexural strength of parts made additively from PETG. To achieve optimization, the GWO algorithm was used, through which the following optimal settings of the manufacturing parameters were identified: deposited layer height 0.20 mm; filling density 100%; extrusion temperature:(230–250) °C; deposition angle 0°. The research presented in [39] focuses on studying the influence of MEX parameters, strain rate and annealing heat treatment on the mechanical properties of additively manufactured parts made of polylactic acid (PLA). The results of the study show that multi-objective optimization is clearly superior to single-objective optimization. The optimal manufacturing parameters are raster angle 90°, strain rate 10^−2^ s^−1^. Annealing heat treatment influences the stiffness, increasing it by up to 15%. In [40], the influence of annealing heat treatment on additively manufactured parts made of acylonitrile butadiene styrene (ABS) is studied, the novelty of the study being represented by the introduction of a new concept consisting of annealing under load (the use of an aluminum mold to limit dimensional deformations during the annealing heat treatment). The results of the study show that annealing at a temperature of 85 °C influences both the tensile strength (increases by 7.6%) and the ductility (decreases). Annealing at 105 °C and 125 °C improves the ductility by 9.7% and 17.56%, respectively, but the impact on the tensile strength is negligible. Annealing under load at a temperature of 105 °C increased the ductility by 19% compared to annealing without load. Annealing under load at a temperature of 125 °C increased the stiffness and a decrease in ductility. The aluminum mold plays a crucial role in reducing dimensional variations, which are reduced by up to 86%. In [41], the influence of the integration of short carbon fibers on the mechanical properties of three-point bending of parts made of PLA is investigated. The study’s conclusions show that by incorporating 6% carbon fibers, improvements of up to 88% in flexural strength are obtained. In [42], the effect of heat treatment on the mechanical properties of additively manufactured PLA parts is examined. Tensile and three-point bending specimens were additively manufactured using different extrusion temperatures, and the specimens manufactured with an extrusion temperature of 210 °C were heat-treated to increase the degree of crystallinity. The results show that increasing the degree of crystallinity of PLA generates an increase in flexural strength by 11–17%. In [43], a study on the influence of annealing treatment on static and dynamic bending responses is presented. The conclusions of the study show that the annealing temperature is the parameter that significantly influences the mechanical properties of PLA additively manufactured specimens. The optimal parameters of the heat treatment are a temperature of 110 °C and duration of 15 min. The paper [44] presents the results of the research on the influence of the parameters of additive manufacturing by material extrusion and the annealing heat treatment on the mechanical properties and tribological characteristics of PEEK. The paper demonstrates that the extrusion temperature and the printing speed are the parameters that significantly influence the fusion between the layers. The heat treatment of the parts increased the tensile strength and compressive strength. At the same time, the annealing heat treatment generated an improvement in the tribological characteristics of the parts, reducing the wear rate. The research presented in [45] investigates the optimization of the thermal post-processing of PLA additively manufactured parts, in order to increase durability. To optimize the heat treatment parameters, the researchers used the Taguchi method and the Grey Relational Analysis, the two analyses leading to the following optimal heat treatment parameters: temperature 90 °C and duration 120 min. The study presented in [46] analyzes the influence of the layer deposited in one pass and the annealing parameters on the tensile strength and dimensional accuracy of parts manufactured by additively manufactured by extrusion of three plastic materials: PLA, PETG, PETG-CF. The results of the study show that high heat-treatment temperatures improve the adhesion between the layers, but cause significant deformations of the parts. In [47], the results of the research on the influence of parameters of additive manufacturing by material extrusion and annealing heat treatment on the compressive strength of specimens made of PETG and recycled PETG are presented. For the study, 90 specimens (45 from PETG and 45 from recycled PETG) were additively manufactured using the following variable parameters of material extrusion: layer height deposited in one pass 0.10–0.20 mm; and percentage filling density 50–100%. All 90 specimens were heat-treated using the following heat treatment parameters: temperature 75 °C and duration 180 min. The results of the study show that after the heat treatment, the compressive strengths of the specimens made of PETG decreased by 31.40%, and the compressive strengths of the specimens made of rPETG increased by 0.63%. In [48], the influence of annealing heat treatment on the mechanical properties of three-point tensile and bending of additively manufactured parts from polylactic acid (PLA) is investigated. The results of the research show that the parts heat-treated for a period of 180 min at a temperature of 75 °C obtained superior mechanical characteristics compared to the parts that were not heat-treated (the tensile strength increased by up to 30%, and the flexural strength increased by 15%). In [49], the influence of annealing heat treatment and the orientation angle of the deposited layers on the tensile strength, hardness and wear is investigated. The conclusions of the study show that the annealing heat treatment resulted in an increase in tensile strengths, an increase in the glass transition temperature and an improvement in the bonding between the layers. In [50], the influence of the annealing heat treatment (temperature, t = 75 °C, duration, d = 180 min) on the tensile properties of the samples manufactured additively from polyethylene terephthalate glycolate (PETG) is investigated. The conclusions of the study show that the annealing heat treatment generated a 10.50% increase in the average breaking strengths of the samples manufactured from PETG.

This study investigates the influence of post-processing by annealing on flexural strength of specimens additively manufactured by extrusion of virgin and recycled polyethylene terephthalate glycol-modified (PETG and rPETG) filaments. The novelty of the work lies in the use of recycled materials in the material extrusion (MEX) additive manufacturing process, the application of annealing heat treatment to improve the mechanical performance of the parts, the comparative analysis of the mechanical performance of the two heat-treated materials, and the application of value analysis as a method for evaluating productivity and rentability.

Using the bibliography used to carry out the bibliographic study in this work and the VOS Viewer software version 1.6.20, the bibliometric map in Figure 1 [51], was drawn.

## 2. Experimental Framework

### 2.1. Design, Additive Manufacturing and Heat Treatment of Three-Point Bending Specimens

The modeling of the three-point bending specimens: the 2D drawing (Figure 2a), the 3D model (Figure 2b) and the STL model was performed in the Solidworks 2023 CAD software according to ISO 178:2019, [52]. The stages of the CAD modeling of the three-point bending specimen are presented in Figure 2 [53].

Using the STL model of the three-point bending specimen, the QIDI Slicer software version 1.2.4 (Figure 3) and the additive manufacturing by material extrusion parameters (Table 1), 3 G-Code files were generated (one file for each layer height, L_h_) containing the work instructions for the additive manufacturing of the three-point bending specimens by extrusion of PETG and rPETG filaments [54].

The 3 G-Code files were loaded onto the QIDI Q1 Pro (QIDI, Wenzhou, China) 3D printer, and subsequently they were run to additively manufacture 45 bending specimens by extrusion of PETG filament (Figure 4a) and 45 bending specimens by extrusion of rPETG filament (Figure 4b). Both filaments (PETG and rPETG) were purchased from Everfil brand (Everfil, Bialystok, Poland). The information from the TDS (technical data sheet) is: diameter: 1.75 ± 0.02 mm, density: 1.29 g/cm^3^ (ISO 1183); Flexural modulus: 2200 MPa (ISO 178); flexural strength: 79 MPa (ISO 178), [52,55].

The 90 three-point bending specimens additively manufactured from virgin and recycled PETG filament (Figure 4) were placed in the Binder ED56 electric oven (Binder GmbH, Tuttlingen, Germany), where they were subjected to the annealing heat treatment. The heat treatment parameters applied to the specimens for the three-point bending test—manufactured using additive technology from PETG and rPETG filament—were a temperature of t = 75 °C and a duration of d = 180 min, followed by slow cooling inside the oven. Annealing heat treatment at 75 °C was conducted just below the glass transition temperature (Tg ≈ 80 °C) of PETG. However, based on the authors’ previous research, this treatment temperature was found to provide the optimal thermal energy to activate polymer chain segment mobility without causing significant deformation. Activating this mobility facilitates the relaxation of internal thermal stresses accumulated during the rapid cooling characteristic of the MEX process and enhances macromolecular diffusion between deposited material layers, thereby closing microscopic voids. Furthermore, the 180 min duration ensures complete thermal stabilization and deep structural relaxation throughout the cross-section of the specimens, directly explaining the observed improvements in mechanical performance.

### 2.2. Determination of the Mechanical Characteristics of Three-Point Bending of Additively Manufactured and Heat-Treated PETG and rPETG Specimens

The specimens manufactured additively by material extrusion of PETG and rPETG filaments and heat-treated were tested for three-point bending in the Materials Technology Laboratory 1, Plastics of the Faculty of Mechanical and Electrical Engineering of the Petroleum Gas University of Ploiesti on the Barrus White 20 kN (Barus, Budapest, Hungary) universal testing machine, according to the ISO 178:2019 standard, using a speed of 5 mm/min [54].

Figure 5 shows the 90 specimens after the three-point bending test on the Barrus White 20 kN universal testing machine.

## 3. Analysis and Interpretation of Results

The results corresponding to the flexural strengths of heat-treated specimens additively manufactured from PETG and rPETG are summarized in Table 2 and Table 3 and Figure 6 and Figure 7, respectively.

According to the results summarized in Table 2 and graphically represented in Figure 6, the maximum average flexural strength of the heat-treated PETG filament additively manufactured specimens (σ_fmax_ = 84.78 MPa) was obtained for the specimens manufactured using L_h_ = 0.20 mm and I_d_ = 100%, and the minimum average flexural strength (σ_fmin_ = 58.51 MPa) was obtained for the specimens manufactured using L_h_ = 0.10 mm and I_d_ = 50%. Increasing the I_d_ value from 50% to 75% increased the average mechanical resistance to bending in 3 points by (7.75–16.18)%, and increasing the Id value from 75% to 100% increased the average mechanical resistance to bending in 3 points by (12.56–17.05)%.

According to the results summarized in Table 3 and graphically represented in Figure 7, the maximum average of flexural strength of the samples additively manufactured from rPETG filament and heat-treated (σ_fmax_ = 74.28 MPa) was obtained for the samples manufactured using L_h_ = 0.20 mm and I_d_ = 100%, and the minimum average flexural strength (σ_fmin_ = 47.80 MPa) was obtained for the samples manufactured using L_h_ = 0.10 mm and I_d_ = 50%. Increasing the I_d_ value from 50% to 75% increased the average flexural strength by up to 8.55%, and increasing the Id value from 75% to 100% increased the average flexural strength by (21.35–27.12)%.

Using the average values of the flexural strength of the additively manufactured PETG and rPETG specimens and heat-treated, the graph in Figure 8 was plotted.

The flexural strengths of the 45 PETG additively manufactured and heat-treated specimens are 14.13% higher than those of the rPETG additively manufactured and subsequently heat-treated specimens.

The flexural strengths of PETG additively manufactured specimens with L_h_ = (0.10/0.15/0.20) mm and I_d_ = 50% and heat-treated are (18.44–22.40)% higher than those of the rPETG specimens using the same parameters.

The flexural strengths of PETG additively manufactured specimens with L_h_ = (0.10/0.15/0.20) mm and I_d_ = 75% and heat-treated are (29.91–33.54)% higher than those of rPETG specimens using the same parameters.

The flexural strengths of PETG additively manufactured specimens with L_h_ = (0.10/0.15/0.20) mm and I_d_ = 100% and heat-treated are (14.13–20.11)% higher than those of rPETG specimens using the same parameters.

The distinct behavior of rPETG can be attributed to the degradation of polymer chains during the recycling process and to structural heterogeneity, which ultimately compromised inter-layer adhesion.

Using Minitab software version 20.1, the variable parameters of additive manufacturing by material extrusion from Table 1, and the results of flexural strengths of the additively manufactured PETG and rPETG specimens (see Table 2 and Table 3), an analysis was performed to evaluate the statistical influence of the variable parameters of additive manufacturing by material extrusion (L_h_ and I_d_) on the flexural strengths. The tools used in the analysis are as follows: ANOVA (ANalysis Of VAriance), Main Effects Plots, DoE (Design of Experiments), Analyse Factorial Design, Contour Plot and Response Optimizer [56].

The results of the ANOVA analysis are summarized in Table 4 and Table 5.

Analyzing the data from Table 4 and Table 5, it can be observed that, for heat-treated specimens made from PETG filament, the infill density percentage—I_d_, (F = 981.25; *p*-Value = 0.000), the layer height—L_h_, (F = 11.48; *p* -Value = 0.002), and the interaction between them (L_h_ · I_d_) (F = 11.26; *p* -Value = 0.002) are all statistically significant. The resulting model explains a substantial proportion of the response variation (R-sq = 96.08%) and demonstrates excellent predictive capability (R-sq pred = 95.16%). For specimens made from rPETG, the influence of infill density, I_d_, is dominant and statistically significant (F = 132.06; *p* -Value = 0.000), while layer height, L_h_, also has a significant effect (F = 11.14; *p* -Value = 0.002). However, in this case, the interaction between the two parameters (L_h_ · I_d_) is statistically insignificant (F = 0.15; *p* -Value = 0.704). The resulting model offers good calibration and high predictive capability (R-sq pred = 74.15%, with R-sq = 77.76%).

The ANOVA analysis demonstrates that the percentage fill density (I_d_) is the critical parameter for both printed materials (PETG and rPETG), exhibiting a dominant statistical influence for PETG (F = 981.25, *p* -Value = 0.000) and rPETG (F = 132.06, *p* -Value = 0.000).

To address statistical uncertainty, Table 5 now presents the standard deviation of the residuals (S) alongside the coefficients of determination (R-sq, R-sq adj, and R-sq pred). The lower standard error for virgin PETG (S = 1.921) compared to recycled PETG (rPETG, S = 4.326) clearly quantifies and reflects the increased dispersion and structural heterogeneity of the recycled polymer matrix.

Following the ANOVA analysis, regression Equations (1) and (2) were generated.

(1) $$\sigma f\;PETG=21.82+110.3\cdot L_{h}\left(mm\right)+0.612\cdot I_{d}\left(\%\right)-1.153\cdot L_{h}\;\left(mm\right)\cdot I_{d}\;\left(\%\right)$$

(2) $$\sigma f\;rPETG=26.30+30.5\cdot L_{h}\left(mm\right)+0.319\cdot I_{d}\left(\%\right)+0.297\cdot L_{h}\;\left(mm\right)\cdot I_{d}\;\left(\%\right)$$

In Figure 9, Pareto graphs were plotted expressing the influence of the variable parameters of additive manufacturing by material extrusion (A = L_h_, B = I_d_ and AB = L_h_ ∙ I_d_) on the flexural strengths of heat-treated specimens fabricated from PETG and rPETG filaments.

An analysis of Figure 9a reveals that, from a statistical standpoint, flexural strength is influenced by parameters A = L_h_, B = I_d_, and AB = L_h_ · I_d_; however, parameter B (I_d_) exerts a dominant influence on the flexural strength of the heat-treated, additively manufactured PETG specimens. The influence of parameter B (I_d_) is 823.89% greater than that of parameter A (L_h_). An analysis of Figure 9b shows that, statistically, flexural strength is influenced by parameters A = L_h_ and B = I_d_; however, parameter B (I_d_) exerts a significantly greater influence on the flexural strength of the heat-treated, additively manufactured PETG specimens compared to parameter A (L_h_). The influence of parameter B (I_d_) is 245.05% greater than that of parameter A (L_h_).

Figure 10 presents the effect diagrams of the factors influencing the flexural performance of specimens additively manufactured from PETG and rPETG filaments and heat-treated.

Analyzing the diagrams in Figure 10, it can be seen how the variable parameters of additive manufacturing by material extrusion [L_h_ = (0.10/0.15/0.20) mm and I_d_ = (50/75/100)%] influence the flexural strengths of specimens manufactured from heat-treated PETG and heat-treated rPETG.

According to Figure 10, increasing the layer height value deposited in one pass and increasing the percentage filling density (I_d_) generates an increase in the flexural strengths of heat-treated specimens, additively manufactured from PETG and rPETG filaments.

In Figure 11, contour plots have been drawn regarding the influence of the parameters L_h_ and I_d_ on the flexural strength of the samples additively manufactured from PETG and rPETG filaments and heat-treated.

Analyzing Figure 11, it is possible to observe the influence of the variable parameters of additive manufacturing by material extrusion (L_h_ and I_d_) on the flexural strengths of the specimens manufactured from PETG and rPETG and heat-treated. According to the contour graphs in Figure 11, it is observed that by increasing the values corresponding to the height of the layer deposited per pass (L_h_) and the percentage filling density (I_d_), the values of the flexural strengths of the specimens manufactured additively from PETG and rPETG filament and subsequently heat-treated are generated.

Using Minitab, the Response Optimizer function, the variable parameters of additive manufacturing (see Table 1) and the values of the flexural strengths (see Table 2 and Table 3), the optimization graphs in Figure 12 were generated.

By analyzing the optimization graphs in Figure 12, it can be concluded that among the nine investigated manufacturing parameter settings [(L_h_ = 0.10/0.15/0.20) mm − I_d_ = (50/75/100)%], the optimal parameters for the additive manufacturing of flexural test specimens via material extrusion using PETG and rPETG filaments are: L_h_ = 0.20 mm and I_d_ = 100%.

## 4. Influence of Heat Treatment on Productivity

Value analysis was used to assess the impact of annealing heat treatment on productivity and economic efficiency, aiming to optimize the ratio between functional utility—defined by flexural strength (V_i_)—and the associated production cost (C_p_).

(3) $$\frac{V_{i}}{C_{p}}\to max,$$

To determine the production cost (C_p_), the following calculation formula was used:

(4) $$C_{p}=Q_{mat}\times P_{mat}+P_{t}\times E_{c}\times P_{en}+A_{t}\times E_{c}\times P_{en},$$

where C_p_ represents production cost (Euro), Q_mat_—the amount of printed material (g) determined with a precision balance, P_mat_—price of PETG and rPETG filaments (Euro/g), P_t_—effective printing time (hours), E_c_—instant printer consumption (kW) measured with a smart plug, P_en_—electricity price (Euro/kWh), and A_t_—annealing time.

The following constant values were used to calculate the production cost C_p_: P_mat-PETG_ = 0.22 EUR/g; P_mat-rPETG_ = 0.19 EUR/g, E_c_ = 0.23 kW/h and P_en_ = 0.32 EUR/kWh. The cost of energy is based on the standard commercial rate, the price of the material is represented by the price actually paid by the authors. The production cost presented in Table 6 was determined using Equation (4). Subsequently, the mean values for the performance metric (flexural strength) were calculated using the 90 values obtained from three-point bending tests on additively manufactured PETG and rPETG parts subjected to heat treatment (PETG-A; rPETG-A). The same procedure was applied to the non-heat-treated three-point bending specimens from the study conducted by the authors in [47].

To determine the return on performance, relationship (3) was used.

(5) $$R_{p}=\frac{\Delta \sigma }{\Delta C}\;,$$

where R_p_ represents return on performance, Δσ—the difference between the average flexural strength of heat-treated specimens and the average flexural strength of untreated specimens, and ΔC—the difference between the production cost of heat-treated samples and the production cost of samples without heat treatment. Adding the data from Table 6 to relationship (5), we obtain:

$$R_{p(PETG)}=\frac{71.33-62.43}{4.34-4.11}=38.70$$

$$R_{p(rPETG)}=\frac{58.10-53.63}{3.75-3.52}=19.73$$

Value analysis demonstrates the efficiency of the heat treatment, evidenced by an increase in the V_i_/C_p_ ratio of 1.25 MPa/Euro and 0.26 MPa/Euro, respectively, compared to untreated reference samples. Furthermore, the cost–benefit indicator confirms that the heat treatment positively impacts productivity, making this post-processing method a viable solution for enhancing mechanical properties.

## 5. Conclusions

This article presents the results of research on the influence of post-processing by annealing three-point bending specimens manufactured additively by material extrusion of PETG and rPETG filaments using the layer height deposited per pass L_h_ = (0.10/0.15/0.20) mm and the percentage filling density I_d_ = (50/75/100)%.

A total of 45 PETG and 45 rPETG specimens were 3D-printed using material extrusion on the QIDI Q1 Pro 3D printer, and subsequently, all 90 specimens were subjected to post-processing by heat treatment with the following parameters: temperature, t = 75 °C and duration, d = 180 min. After completing post-processing, all 90 specimens were tested for three-point bending on the Barrus White machine, which has a capacity of 20 kN.

In the case of PETG additively manufactured specimens and subsequently heat-treated, the arithmetic mean is 71.33 MPa, the maximum mean value σ_fmax_ = 84.78 MPa was obtained for the set of specimens manufactured with L_h_ = 0.20 mm − I_d_ = 100%, and the minimum mean value σ_fmin_ = 58.51 MPa was obtained for the set of specimens manufactured with L_h_ = 0.10 mm − I_d_ = 50%. The sequential increase in the I_d_ value from 50% to 75% and from 75% to 100% determined the increase in flexural strengths by (7.76–16.18)% and (12.56–17.05)%, respectively.

In the case of additively manufactured samples from rPETG and subsequently heat-treated, the arithmetic mean is 58.10 MPa, the maximum average value σ_fmax_ = 74.28 MPa was obtained for the set of samples manufactured with L_h_ = 0.20 mm − I_d_ = 100%, and the minimum average value σ_fmin_ = 47.80 MPa was obtained for the set of samples manufactured with L_h_ = 0.10 mm − I_d_ = 50%. The sequential increase in the I_d_ value from 50% to 75% and from 75% to 100% determined the increase in flexural strengths by 8.55% and (21.35–27.12)%, respectively.

The results of the flexural strengths of the additively manufactured PETG and rPETG specimens and heat-treated are 14.24% higher in the case of PETG and 2.48% higher in the case of rPETG compared to those of the specimens manufactured from PETG and rPETG using the same material, manufacturing parameters, testing condition, but not heat-treated [47]. Based on the results obtained, it can be concluded that the annealing heat treatment had a beneficial effect on the three-point bending specimens, improving their mechanical strength; the polymers were not damaged, and no deformation of the parts was observed.

The results of this study demonstrate that the use of post-processing by annealing heat treatment generates superior mechanical properties of the additively manufactured PETG and rPETG specimens.

In future works, the authors aim to extrapolate the study to other types of mechanical tests and the characterization of the materials by performing SEM, DSC and TGA analyses. At the same time, the authors aim to study the influence of annealing heat treatment on dimensional accuracy.

## Figures and Tables

**Figure 1:**
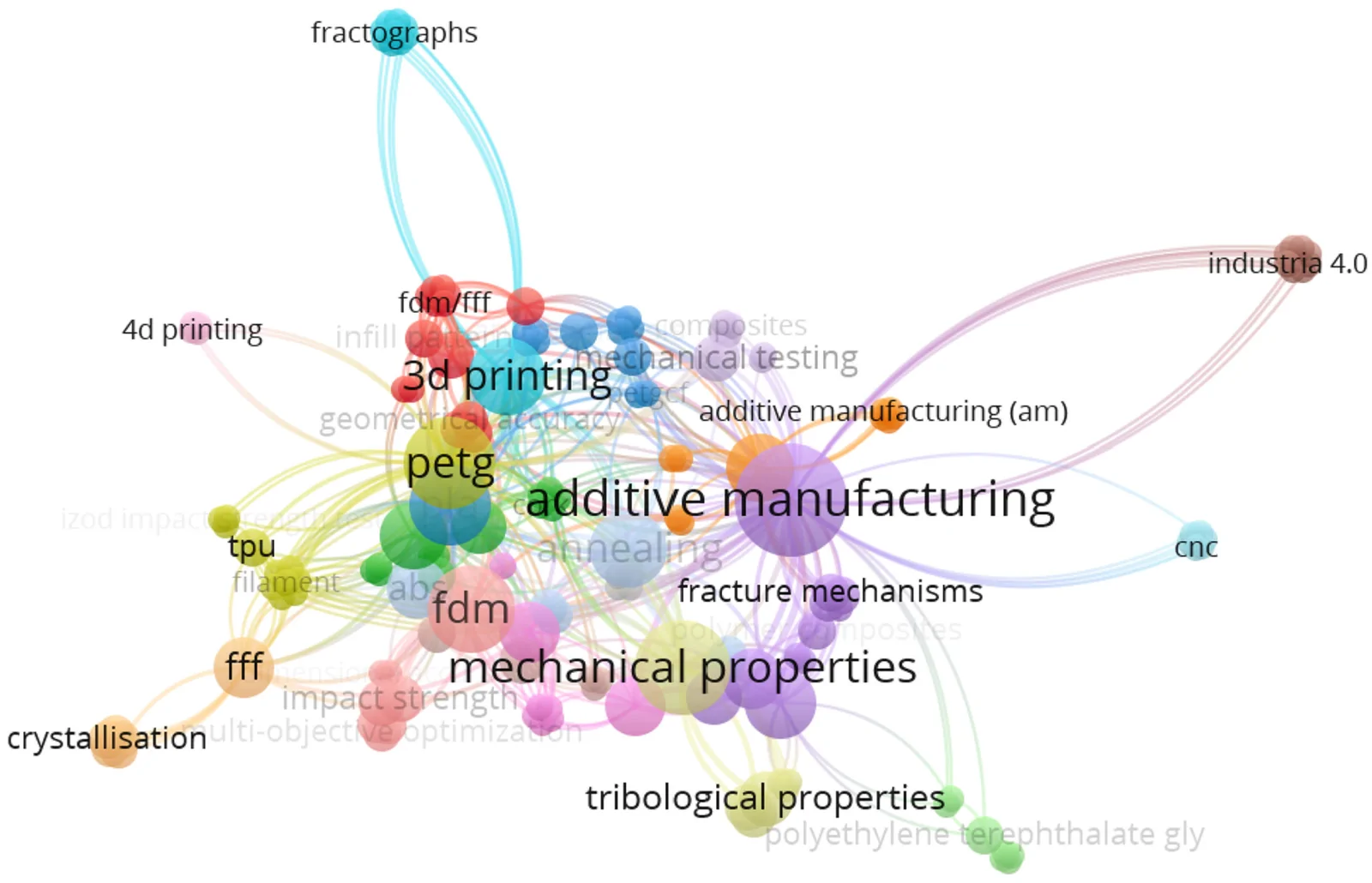
Keyword co-occurrence network from the bibliography.

**Figure 2:**
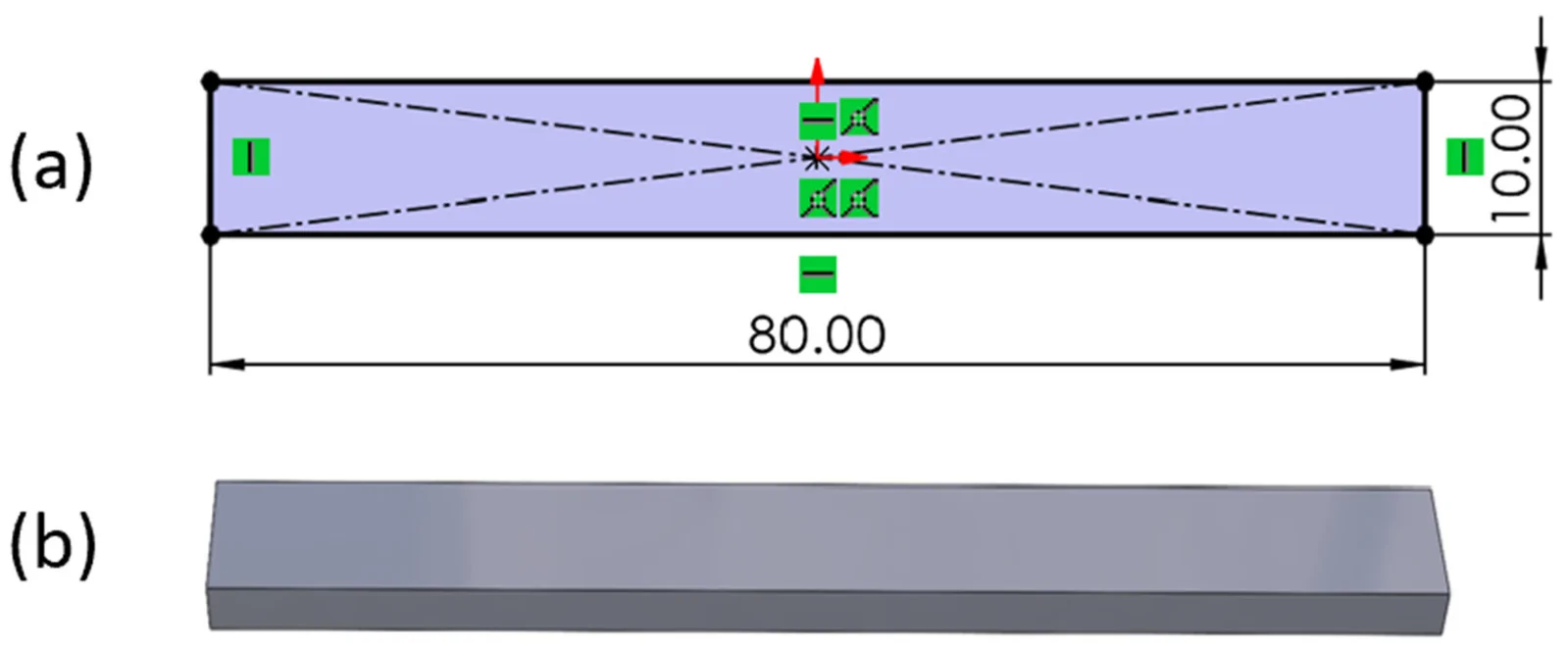
Steps of CAD modeling of the three-point bending specimen: (**a**) 2D sketch; (**b**) 3D model.

**Figure 3:**
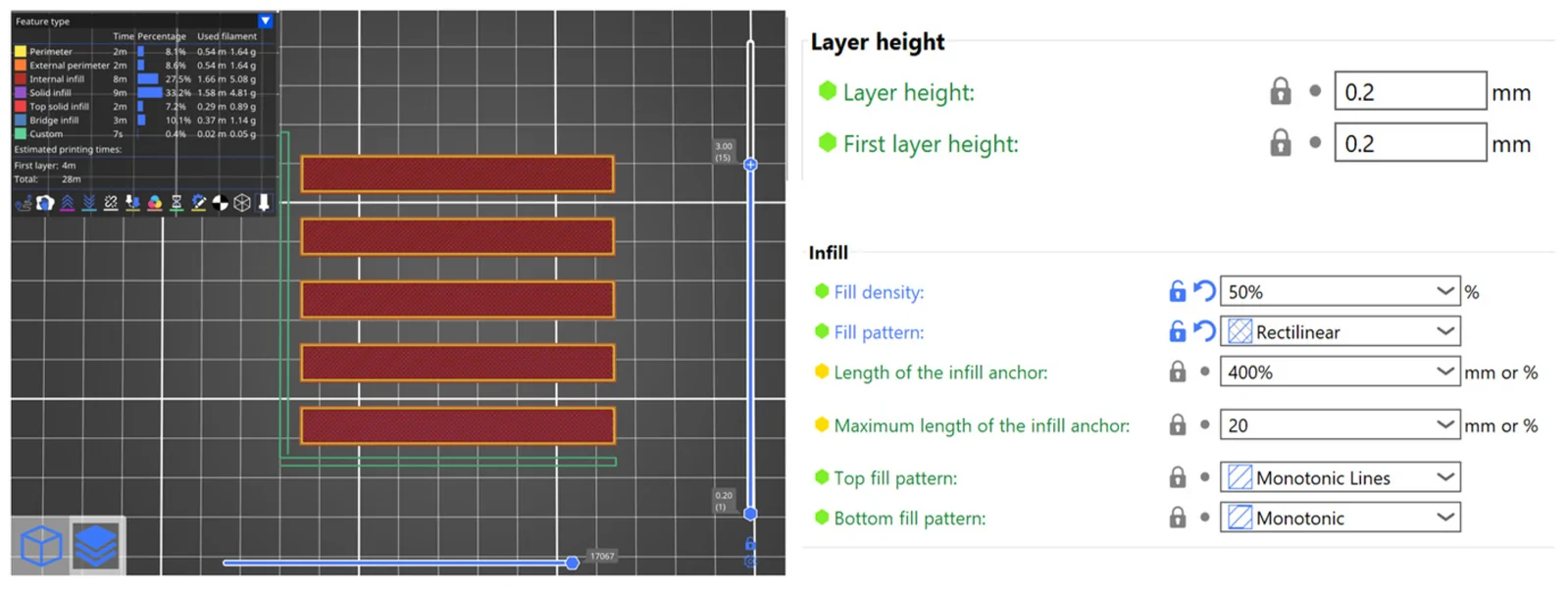
Setting working parameters and generating G-Code files for additive manufacturing of three-point bending specimens.

**Figure 4:**
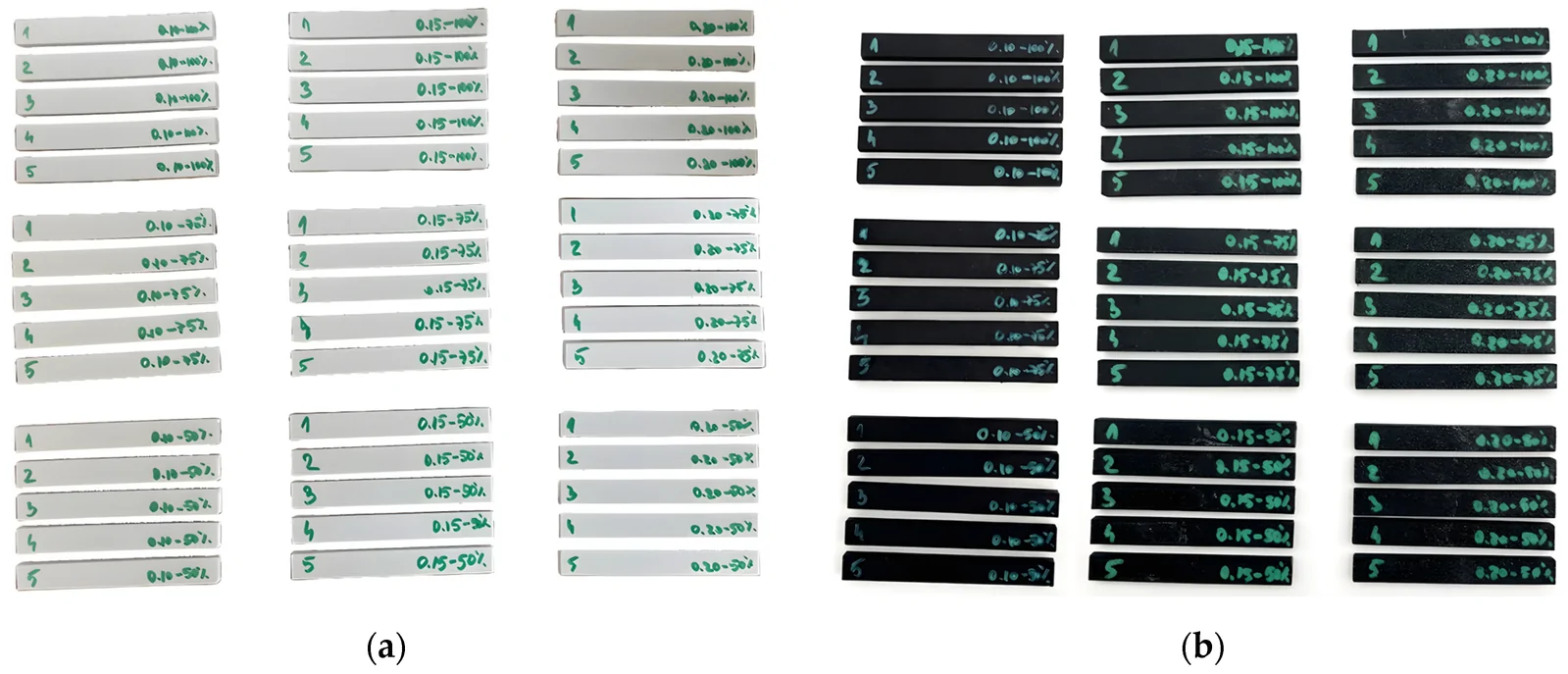
Three-point bending specimens additively manufactured on the QIDI Q1 Pro printer: (**a**) PETG; (**b**) rPETG.

**Figure 5:**
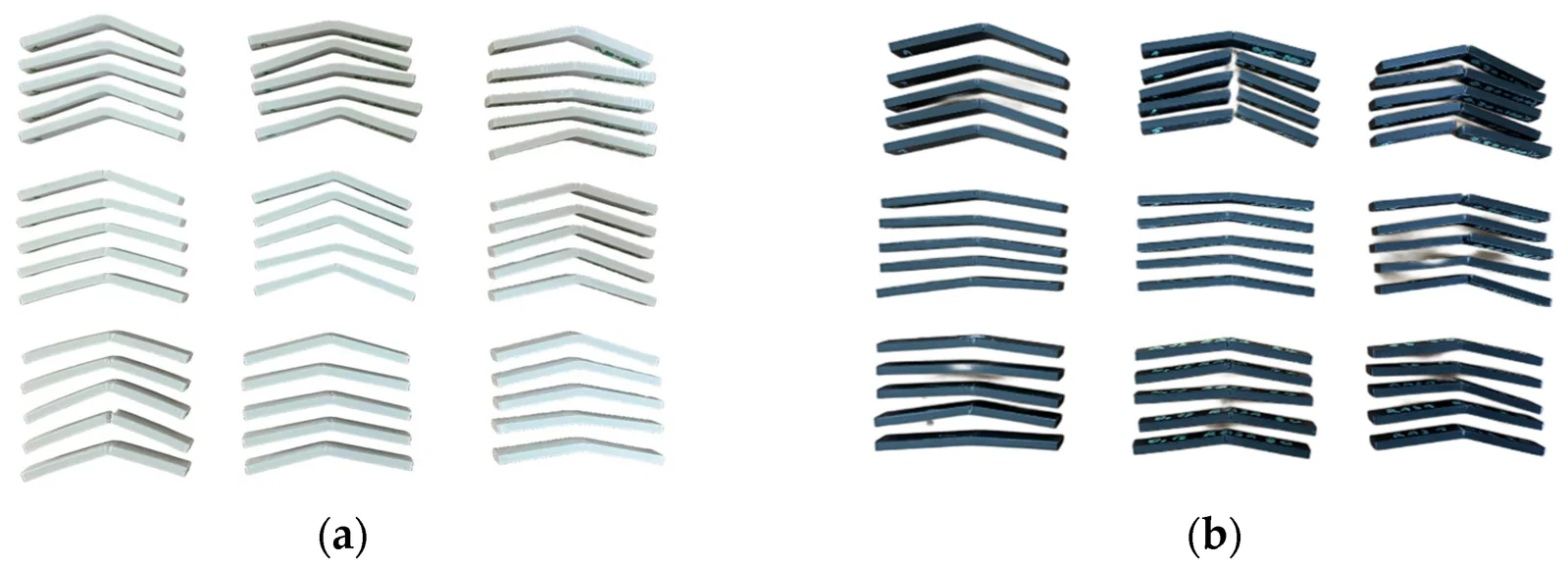
Specimens manufactured on the QIDI Q1 Pro printer after three-point bending testing on the Barrus White 20 kN machine: (**a**) heat-treated PETG; (**b**) heat-treated rPETG.

**Figure 6:**
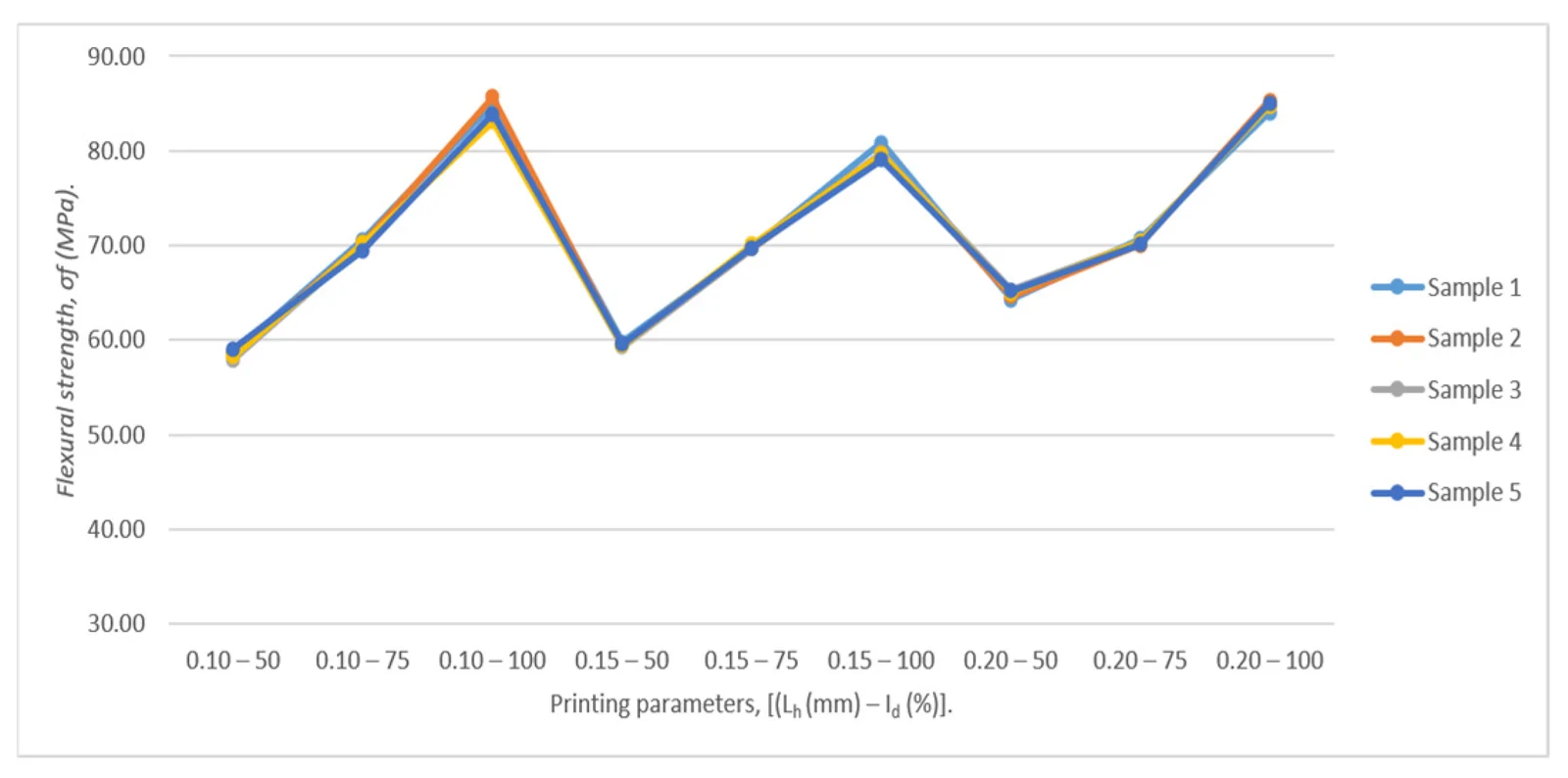
Graphical representation of the flexural strengths of specimens made of PETG and heat-treated.

**Figure 7:**
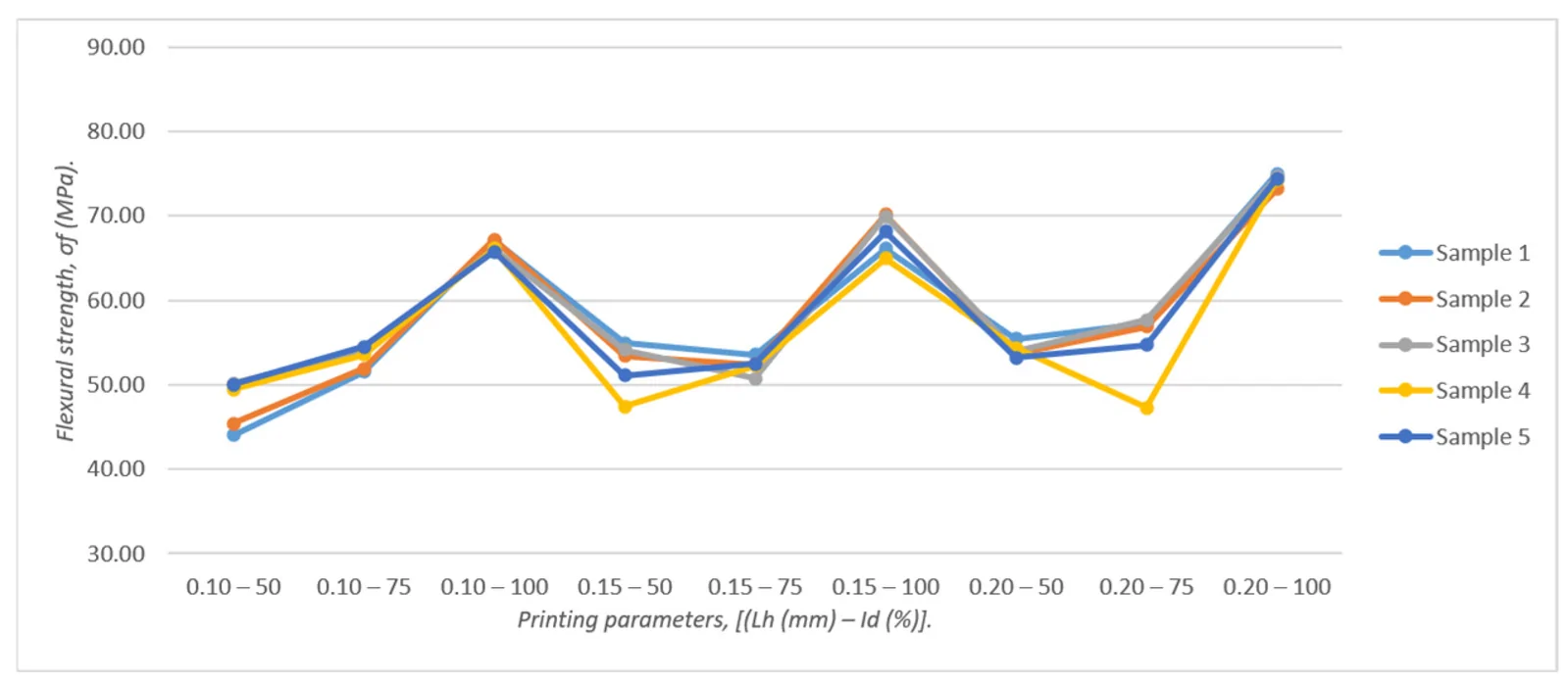
Graphical representation of the flexural strengths of specimens manufactured from rPETG and heat-treated.

**Figure 8:**
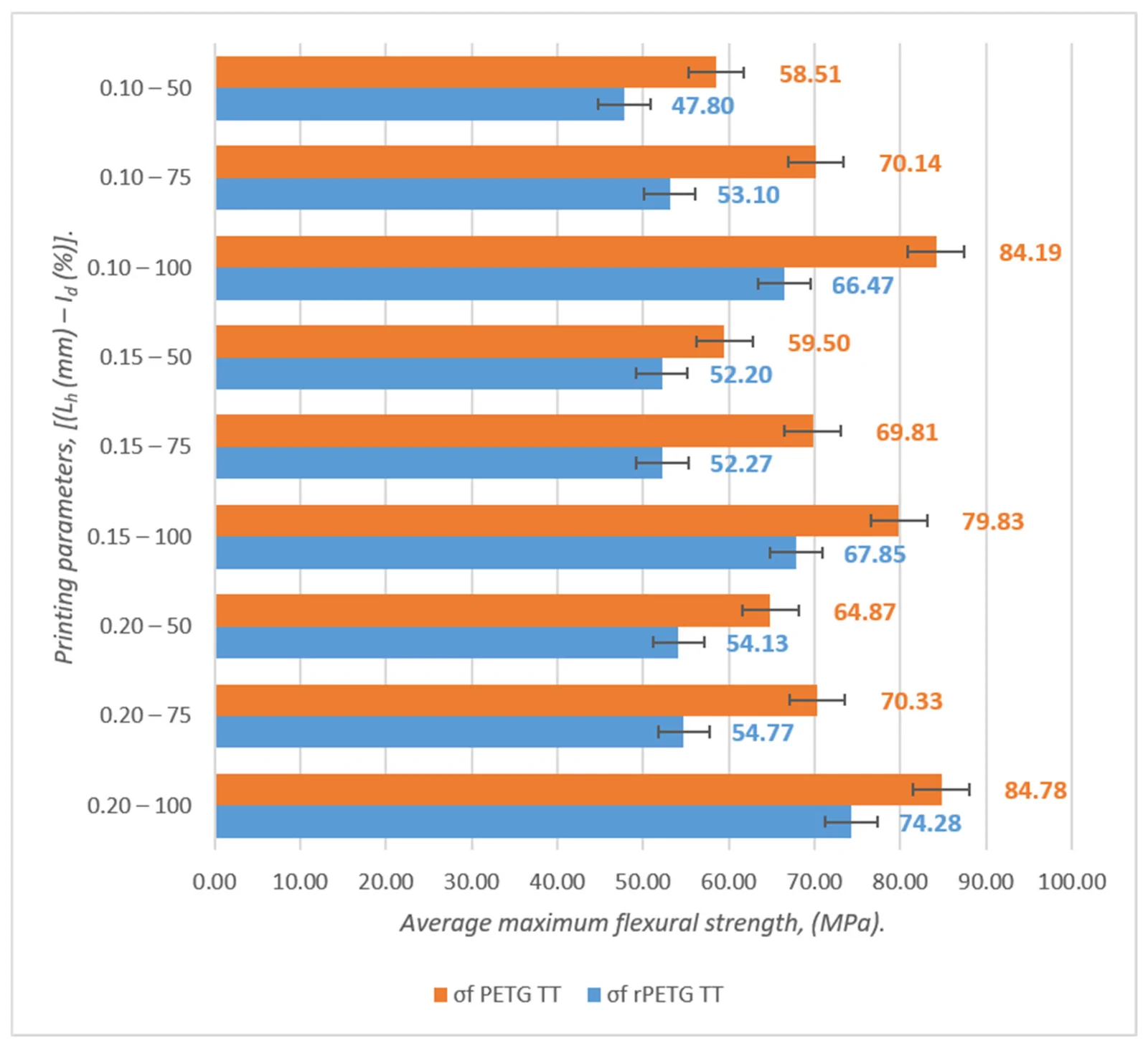
Average maximum values of flexural strengths of additively manufactured PETG and rPETG specimens and heat-treated.

**Figure 9:**
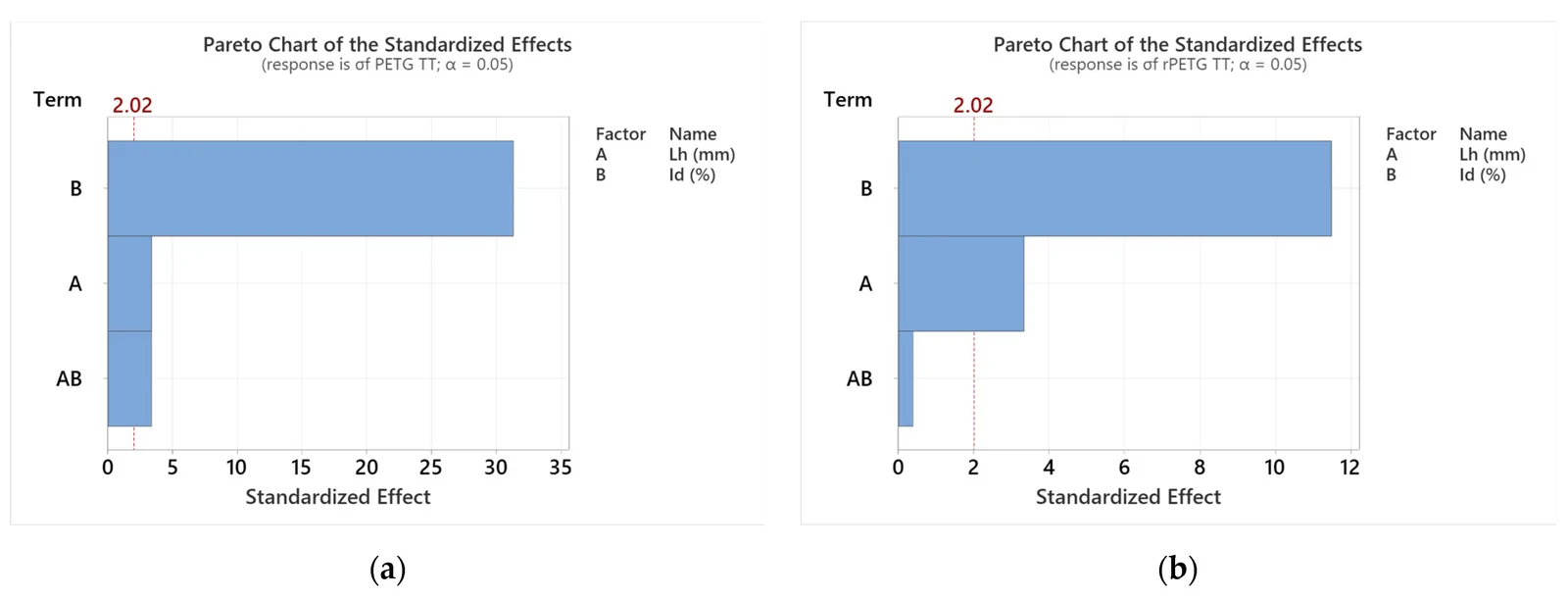
Influence of variable (A = L_h_; B = I_d_ and AB = L_h_ ∙ I_d_) of additive manufacturing by material extrusion on the mechanical flexural strengths of heat-treated: (**a**) heat-treated PETG; (**b**) heat-treated rPETG.

**Figure 10:**
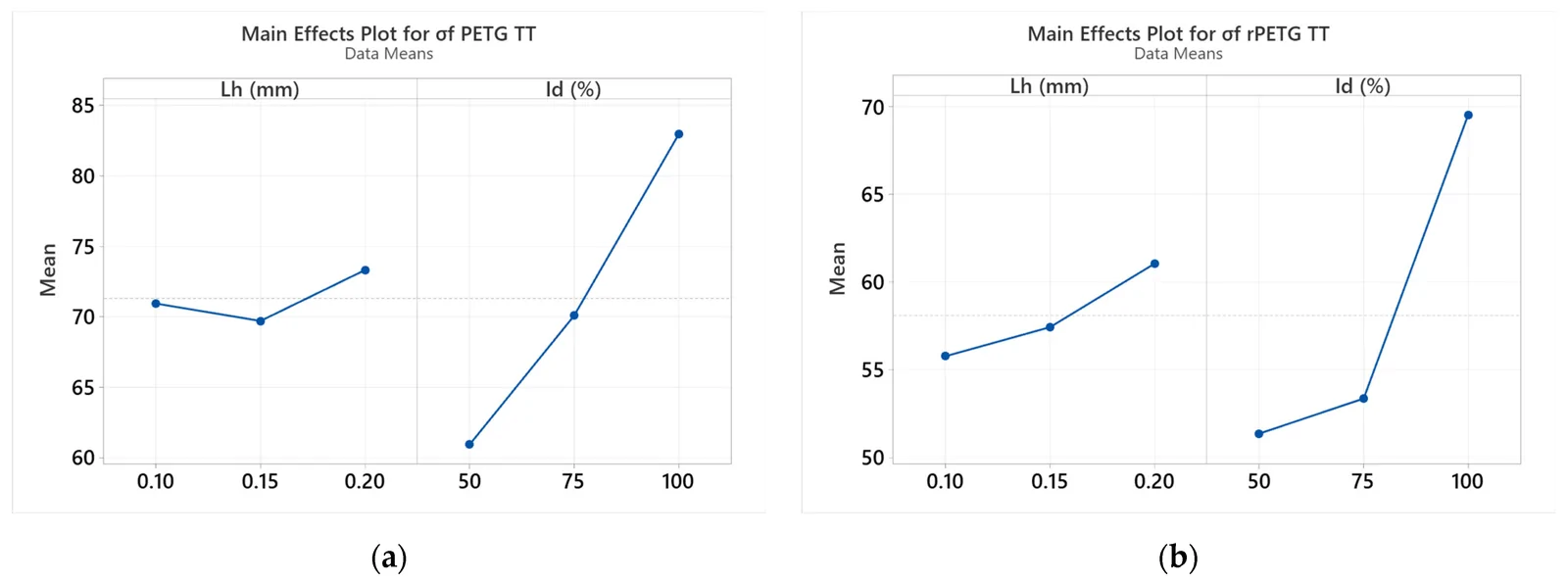
Effect diagrams of factors influencing the flexural strengths of specimens manufactured from: (**a**) heat-treated PETG; (**b**) heat-treated rPETG.

**Figure 11:**
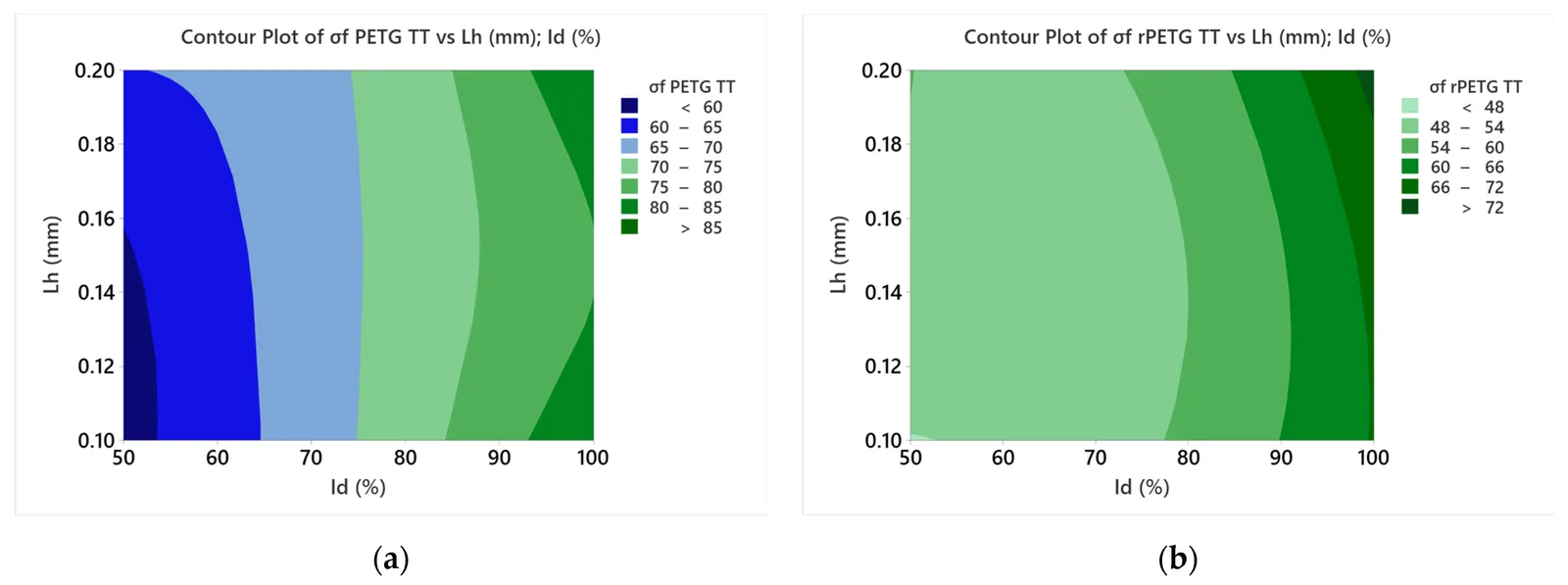
Contour plots on the influence of variable parameters of additive manufacturing by material extrusion on the flexural strengths of additively manufactured specimens from: (**a**) heat-treated PETG; (**b**) heat-treated rPETG.

**Figure 12:**
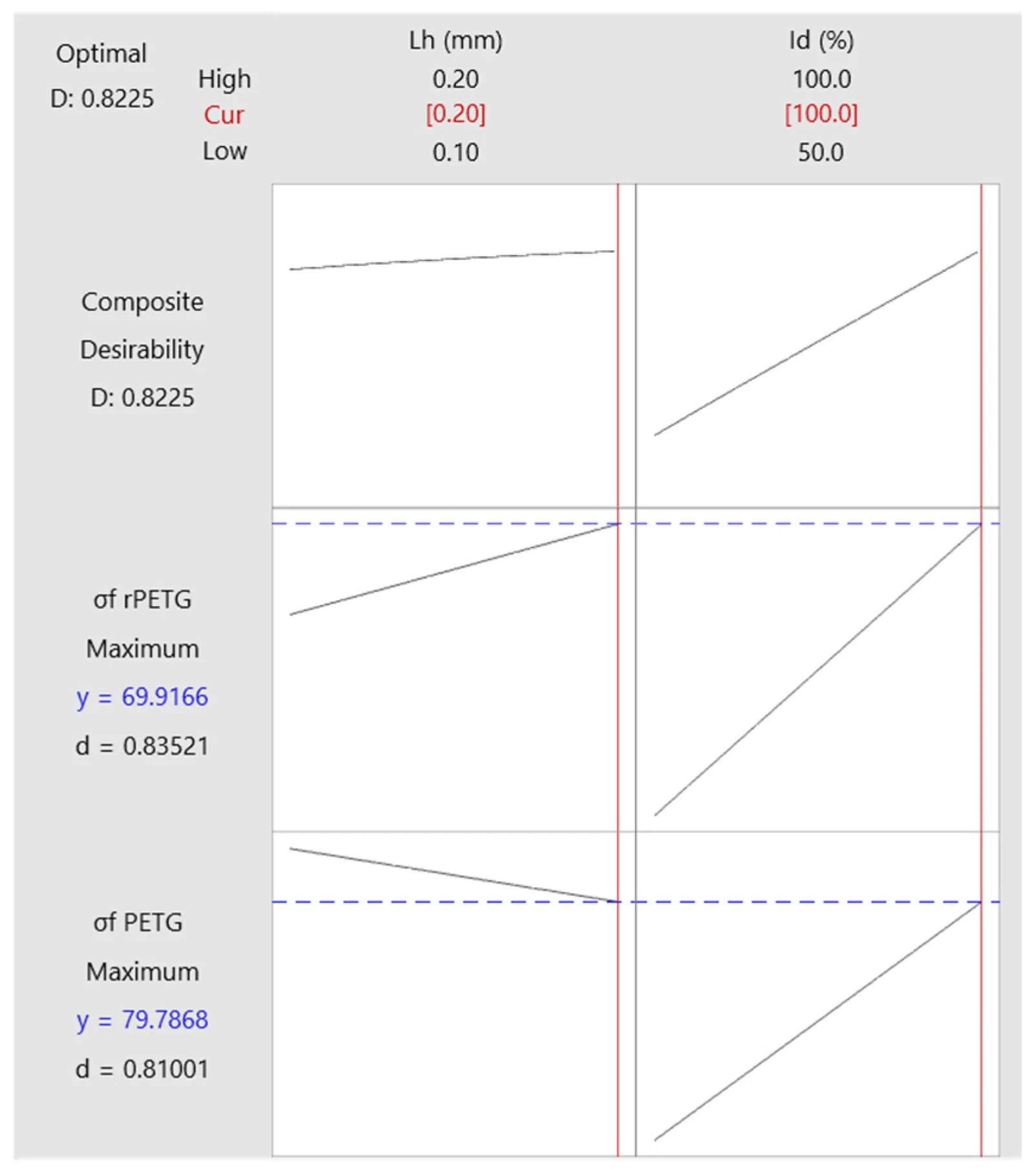
Manufacturing parameter optimization plots for three-point bending specimens of heat-treated PETG and heat-treated rPETG.

**Table 1:** Additive manufacturing parameters by material extrusion for the manufacture of three-point bending specimens from PETG and rPETG.

Printing Parameters	PETG; rPETG
Orientation, O	Flat X-Y
Extruder temperature, E_t_	250 °C
Platform temperature, P_t_	70 °C
Printing speed, P_s_	120 mm/s
Plate adhesion, P_a_	Brim
Travel speed, T_s_	500 mm/s
Vertical travel speed, V_ts_	10 mm/s
Layer height, L_h_	0.10/0.15/0.20 mm
First layer height, F_lh_	0.20 mm
Perimeters, P	2
Top solid layers, T_sl_	5
Bottom solid layers, B_sl_	2
Seam position, S_p_	Aligned
Filling density percentage, I_d_	50/75/100%
Infill pattern, I_p_	Rectilinear
Raster angle, R_a_	45°
Top fill pattern, T_f_	Monotonic lines
Bottom fill pattern, B_f_	Monotonic
Extrusion multiplier, E_m_	0.95
Pressure advance, P_a_	0.086 mm/s
Nozzle diameter, N_d_	0.40 mm

**Table 2:** The values of flexural strength of samples additively manufactured from PETG filament and heat-treated.

Layer Height, L_h_	Infill Density, I_d_	Maximum Flexural Strength, σ_f_					Average σ_f_
(mm)	(%)	Sample Number					(MPa)
		1	2	3	4	5	
0.10	50%	58.72	58.61	57.90	58.25	59.08	**58.51**
	75%	70.61	70.25	70.14	70.26	69.43	**70.14**
	100%	84.97	85.67	83.32	83.08	83.91	**84.19**
0.15	50%	59.78	59.43	59.31	59.43	59.55	**59.50**
	75%	69.78	69.67	69.67	70.14	69.78	**69.81**
	100%	80.85	79.79	79.79	79.67	79.08	**79.83**
0.20	50%	64.25	64.61	65.31	64.96	65.20	**64.87**
	75%	70.73	70.02	70.37	70.37	70.14	**70.33**
	100%	84.02	85.32	84.73	84.73	85.08	**84.78**

**Table 3:** Mechanical resistance values for three-point bending of samples additively manufactured from rPETG filament and heat-treated.

Layer Height, L_h_	Infill Density, I_d_	Maximum Flexural Strength, σ_f_					Average σ_f_
(mm)	(%)	Sample Number					(MPa)
		1	2	3	4	5	
0.10	50%	44.01	45.42	50.13	49.43	50.01	**47.80**
	75%	51.54	51.90	54.02	53.54	54.49	**53.10**
	100%	67.08	67.20	66.14	66.14	65.78	**66.47**
0.15	50%	54.96	53.43	54.13	47.43	51.07	**52.20**
	75%	53.54	52.37	50.72	52.25	52.49	**52.27**
	100%	66.14	70.14	69.90	64.96	68.14	**67.85**
0.20	50%	55.43	53.66	54.02	54.37	53.19	**54.13**
	75%	57.31	56.84	57.66	47.31	54.72	**54.77**
	100%	74.96	73.20	74.61	74.26	74.37	**74.28**

**Table 4:** ANOVA statistical findings.

Material	Source	Degrees of Freedom, (DF)	Adjusted Sum of Squares, (Adj SS)	Adjusted Mean Square, (Adj MS)	F-Value	*p*-Value
PETG	L_h_ (mm)	1	42.38	42.38	11.48	0.002
	I_d_ (%)	1	3621.66	3621.66	981.25	0.000
	L_h_ · I_d_	1	41.57	41.57	11.26	0.002
	Error	41	151.32	3.69	-	-
	Total	44	3856.94	-	-	-
rPETG	L_h_ (mm)	1	208.45	208.45	11.14	0.002
	I_d_ (%)	1	2471.77	2471.77	132.06	0
	L_h_ · I_d_	1	2.75	2.75	0.15	0.704
	Error	41	767.43	18.72	-	-
	Total	44	3450.40	-	-	-

**Table 5:** Model performance and validation indicators.

Material	S	R-sq	R-sq (adj)	R-sq (pred)
PETG	1.921	96.08%	95.79%	95.16%
rPETG	4.326	77.76%	76.13%	74.15%

**Table 6:** Determination of the V_i_/C_p_ ratio.

Material	V_i_ (MPa)	C_p_ (Euro)	V_i_/C_p_ (MPa/Euro)
PETG	62.43	4.11	15.19
rPETG	53.63	3.52	15.24
PETG-A	71.33	4.34	16.44
rPETG-A	58.10	3.75	15.49

## Data Availability

Data are contained within the article. The data presented in this study are available upon request from the corresponding author.
